# The role of senescent T cells in immunopathology

**DOI:** 10.1111/acel.13272

**Published:** 2020-11-09

**Authors:** Luciana P. Covre, Roel P. H. De Maeyer, Daniel C. O. Gomes, Arne N. Akbar

**Affiliations:** ^1^ Division of Medicine University College London London UK; ^2^ Núcleo de Doenças Infecciosas Universidade Federal do Espírito Santo Vitoria Brazil

**Keywords:** aging, senescence, T cell

## Abstract

The development of senescence in tissues of different organs and in the immune system are usually investigated independently of each other although during ageing, senescence in both cellular systems develop concurrently. Senescent T cells are highly inflammatory and secrete cytotoxic mediators and express natural killer cells receptors (NKR) that bypass their antigen specificity. Instead they recognize stress ligands that are induced by inflammation or infection of different cell types in tissues. In this article we discuss data on T cell senescence, how it is regulated and evidence for novel functional attributes of senescent T cells. We discuss an interactive loop between senescent T cells and senescent non‐lymphoid cells and conclude that in situations of intense inflammation, senescent cells may damage healthy tissue. While the example for immunopathology induced by senescent cells that we highlight is cutaneous leishmaniasis, this situation of organ damage may apply to other infections, including COVID‐19 and also rheumatoid arthritis, where ageing, inflammation and senescent cells are all part of the same equation.

## Graphical abstract

Senescence occurs in tissues as well as in the immune system but the interaction between this is poorly understood. Senescent T cells are pro‐inflammatory, express NK receptors that can act independent of the TCR, and are highly cytotoxic. They recognise stress ligands expressed by cells in inflamed or infected tissues or as a consequence of cellular senescence. Here, we discuss how senescent T cells might contribute to exacerbated tissue damage in infectous contexts by interacting with senescent stromal cells. We focus on cutaneous leishmaniasis as a model for this but suggest that this occurs in other infections such as COVID‐19 and indeed autoimmune diseases such as rheumatoid arthritis.

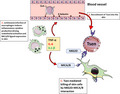

## 1 INTRODUCTION

As we age, we accumulate cells in many organs that exhibit signs of DNA damage, have poor proliferative capacity and are highly secretory. These cells are senescent, defined as being in a state of cell cycle arrest associated with phenotypic and functional changes (Campisi, 2013; Campisi & Fagagna, 2007). This process is primarily thought to prevent cancerous transformation of dividing cells (Braig et al., 2005; Chen et al., 2005), but senescence plays a vital role in developmental biology as well as in wound healing/tissue repair (Demaria et al., 2014; Krizhanovsky et al., 2008; Kurundkar et al., 2016; Pitiyage et al., 2011; van Deursen, 2014). While transient senescence is a beneficial mechanism earlier in life, the accumulation of senescent cells with increasing age leads to organ dysfunction, driving inflammation and may underlie many age‐related diseases such as atherosclerosis (Childs et al., 2016), osteoarthritis (Jeon et al., 2017), neurodegenerative diseases (Chinta et al., 2018; Kritsilis et al., 2018) and cirrhosis (Wiemann et al., 2002).

Senescence is triggered by activation and subsequent maintenance of DNA damage repair (DDR) signalling pathways. This can by induced by oncogene activation, replicative stress related to telomere erosion and oxidative stress (Allsopp, 2008; Blackburn et al., 2015) that kickstart a DDR. This involves proteins such as γ‐H2AX, ATM, ATR, p16 and p53 (Nassour et al., 2016) that leads to cycle arrest which becomes permanent if repair fails. Importantly, while senescent cells do not proliferate, they are still metabolically active and secrete a wide array of cytokines, chemokines and matrix metalloproteinases that vary according to the cell type involved (Coppé et al., 2010). It is this senescence‐associated secretory phenotype (SASP) that is thought to play a crucial role in age‐related pathology such as the chronic low grade inflammation observed with advanced age, called inflammageing (Franceschi et al., 2000). Therefore, two recognized characteristics of senescent cells are the lack of proliferative activity and the presence of a senescence‐associated secretory phenotype (SASP, Campisi, 2013).

While senescence was first discovered in fibroblasts and extensively worked on in other non‐leukocytic cells, it has become increasingly clear that immune cells undergo senescence as well. Within the immune system, the existence of non‐proliferative leukocyte populations that have high capacity for biologically active mediator secretion has been recognized for many decades, albeit under a different name. These are the effector T lymphocytes that secrete pro‐inflammatory cytokines and cytotoxic granules but do not proliferate after activation (Dunne et al., 2005). Recent studies show that these cells also harbour DNA damage, short telomeres, low telomerase activity and engage signalling pathways associated with cellular senescence (Callender et al., 2018; Henson et al., 2014; Lanna et al., 2014). Therefore, the terms effector T cells and senescent T cells may be synonymous and refer to the same T cell populations. Effector T cells that are expanded in number during the acute phase of a human anti‐viral response to Epstein‐Barr Virus (EBV) are highly expanded in number and lose the expression of the anti‐apoptotic molecule Bcl‐2 that makes them short‐lived (Akbar et al., 1993). These cells also have relatively long telomeres due to upregulation of the enzyme telomerase and are in cell cycle (Maini et al., 1999). The difference betwen such short‐lived effector cells and senescent T cells in the steady state is that the latter are non‐proliferative, do not express telomerse after activation, exhibit short telomeres and express senescence related signalling molecules as described above. Nevertheless senescent cells in the steady state in both CD4 and CD8 compartments still express lower levels of Bcl‐2 that the other subsets. Collectively this indicates that the extent of T cell proliferation the acute phase of a viral infection drives T cells to senescence. These cells are still susceptible to apotosis but can persist if sufficient antiapototic cytokines are tissue niches. It can be argued that senescent T cells derive from a subpopulation of effector T cells that do not undergo apoptosis, instead becoming senescent and lingering long term. In this review, we discuss recent observations suggesting that certain infectious agents can drive the accumulation of CD8^+^ T cells that exhibit all the hallmarks of senescence as described above (henceforth referred to as T_sen_). We will focus on patients infected with *Leishmania braziliensis* where the increased T_sen_ numbers in the skin may induce the characteristic skin lesions associated with this disease. We propose a hypothesis, bases on existing data, that this pathology occurs because senescent non‐lymphoid cells in the skin are be killed by infiltrating T_sen,_ a novel interaction between senescent lymphoid and non‐lymphoid cells that may also have implications for ageing.

## 2 FUNCTIONAL PROPERTIES OF CD8^+^ T_sen_

CD8^+^ T cells can be subdivided based on expression of the co‐stimulatory molecules CD27 and CD28, where naïve CD8^+^ T cells co‐express both markers and as they differentiate to an effector phenotype lose CD28 expression and subsequently CD27 (Henson et al., 2012). Sometimes these will be subdivided further into T_EMRA_ cells, so named because they re‐express CD45RA (Henson et al., 2012). The CD27^−^CD28^−^ compartment harbours T_sen_ cells which can be identified further by expression of KLRG1 and CD57 (Brenchley et al., 2003; Henson et al., 2009). Moreover, many studies have shown a plethora of markers that are upregulated on T_sen_ cells, such as downregulated telomerase, shortened telomeres, DNA damage responses and constitutive MAPK activity, features specific to T_sen_ over effector T cells. T_sen_ also exhibit a SASP consisting of proteases and pro‐inflammatory mediators such as TNF and IL‐1β (Callender et al., 2018). These functional changes are summarized in Table 1 (Akbar et al., 1993; Brenchley et al., 2003; Callender et al., 2018, 2019; Geginat et al., 2003; Gumá et al., 2004; Henson et al., 2009, 2012, 2014, 2015; Libri et al., 2011; Maini et al., 1999; Ouyang et al., 2003; Pereira et al., 2020; Plunkett et al., 2001; Tarazona et al., 2001, 2002; Voehringer et al., 2002; Weng et al., 2009). Single cell RNAseq analysis of peripheral blood derived CD8^+^ T cells show that CD8^+^ T cells identified by the above functional criteria also express multiple features associated with cellular senescence (Table 1, Pereira et al., 2020). This indicates that the terms senescent and effector CD8^+^ T cells are synonymous and identify identical populations.

**Table 1:** List of T cell, NK cell and senescence‐associated marker expression in CD8 T cell subsets

Cellular feature	Naïve	Central memory	Effector memory	EMRA/senescent	References
T cell‐associated					
CD27	+++	++	−	−	Henson et al. (2012)
CD28	+++	++	+/−	−	Henson et al. (2012)
CD45RA	+++	++	−	++	Henson et al. (2012)
CCR7	+++	++	−	+/‐	Henson et al. (2012)
CD62L	++	++	−	−	Henson et al. (2012)
Lck‐LAT‐Zap70	+	+	+	+/−	Pereira et al. (2020)
PI3 K‐Akt‐mTOR	+	+	+	+/−	Henson et al. (2014)
TCR‐mediated proliferation	+++	+++	++	+/−	Henson et al. (2009) Pereira et al. (2020)
NK cell‐associated					
CD57	−	−	+	++	Brenchley et al. (2003)
KLRG1	−	−	+	++	Voehringer et al. (2002) Ouyang et al. (2003) Henson et al. (2009)
NKG2A/C	−	−	+/‐	+	Tarazona et al. (2002) Gumá et al. (2004) Pereira et al. (2020)
NKG2D	+	+	+	++	Tarazona et al. (2002) Gumá et al. (2004)
CD244	+	−	−	+++	Tarazona et al. (2002)
KIRs	−	−	−	++	Gumá et al. (2004) Pereira et al. (2020)
DAP12	−	−	−	+	Pereira et al. (2020)
Senescence					
Proliferative capacity	+++	+++	++	−	Weng et al. (2009)
Telomere length	+++	+++	++	+/−	Plunkett et al. (2001) Weng et al. (2009)
Telomerase	+++	++	+	−	Maini et al. (1999) Weng et al. (2009)
BCL‐2	+++	++	+	+/−	Akbar et al. (1993) Geginat et al. (2003) Libri et al. (2011)
p16	−	−	−	+	Henson et al. (2015) Pereira et al. (2020)
γH2AX	−	−	−	++	Callender et al. (2019)
DDR	−	−	−	+	Callender et al. (2018) Pereira et al. (2020)
SASP	−	−	−	++	Callender et al. (2018)
IL‐1β	−	−	++	+	Callender et al. (2018)
IL‐18	−	−	−	++	Callender et al. (2018)
CCL16	−	−/+	++	+++	Callender et al. (2018)
ADAM28	−	−	−	+++	Callender et al. (2018)
Sestrins	−	−	+	++	Pereira et al. (2020)
MAPK (p38/Erk/Jnk)	−	−	+/−	++	Henson et al. (2014), Henson et al. (2015) Pereira et al. (2020)

CD8^+^ T_sen_ have limited proliferative capacity after activation via the T cell receptor (TCR) complex (Akbar et al., 2016) that is due in part to downregulation of key TCR signalling molecules such as LCK, LAT and PLC‐γ (Pereira et al., 2020). CD4^+^ T_sen_ also exhibit identical markers of senescence to their CD8^+^ counterparts (Lanna et al., 2014) but are present at much lower frequency that CD8^+^ T_sen_ in the peripheral blood of healthy donors. The development of senescence characteristics in T cells was initially considered to indicate the dysfunction of these cells during ageing (Akbar et al., 2004). However, CD8^+^ T_sen_ populations express surface receptors that are associated with NK cells such as NKG2D, NKG2C, NKG2A and Killer immunoglobulin‐like receptor (KIR) families compared to undifferentiated and non‐senescent CD28^+^CD27^+^ CD8^+^ T cells (Abedin et al., 2005; Michel et al., 2016; Pereira et al., 2020; Vallejo et al., 2011). This suggests rather than being dysfunctional, these cells acquire an alternative functional profile as they differentiate towards senescence. This is supported by observations that these T_sen_ express DAP12, an NK cytotoxicity adaptor molecule, and are capable of killing tumour cell lines in an MHCI‐independent manner (Pereira et al., 2020). Mechanistically, the switch from TCR to NKR expression in CD8^+^ T_sen_ is regulated by stress proteins known as the sestrins in both humans and mice (Pereira et al., 2020). Of note, CD4^+^ T_sen_ also express NKRs, suggesting that they may also mediate effector functions through these receptors (Pereira et al., 2020).

**Figure 1:**
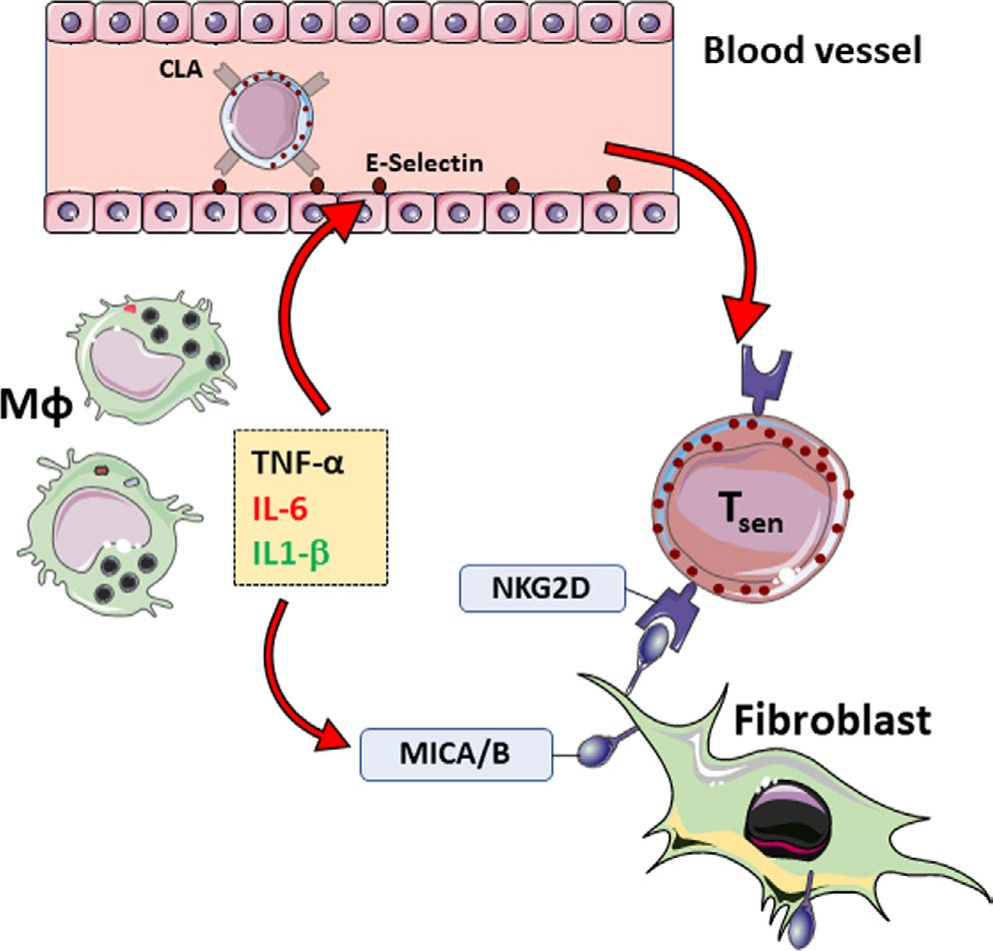
Leishmania infection results in infiltration of T_sen_ that aberrantly kill stressed and senescent stromal cells contributing to excessive damage. Leishmania infection of macrophages induces inflammatory cytokine production driving expression of MICA/B and other stress ligand on resident skin cells such as fibroblasts. Concurrently, these cytokines activate the endothelium which upregulates adhesion markers like E‐selection. This binds to CLA expressing senescent T cells enabling their infiltration into the skin. Here, these senescent T cells, expressing a host of NKRs including NKG2D interact with resident skin cells. The interaction between NKG2D^+^ T_sen_ and MICA/B^+^ fibroblasts results in killing of the latter and contributing to off‐target tissue pathology.

## 3 CD8^+^ T_sen_ ACCUMULATE DURING AGEING AND PERSISTENT VIRAL INFECTION

Previous reports have shown that CD8^+^ T_sen_ accumulate in older humans and that this is largely driven by ongoing sub‐clinical responses to persistent viruses especially cytomegalovirus (CMV) (Jackson et al., 2017; Nikolich‐Žugich et al., 2020; Savva et al., 2013). Moreover, this population has increased capacity to rapidly respond to signals mediated by inflammatory cytokines (Freeman et al., 2012). Furthermore, proinflammatory cytokine production associated with persistent CMV infection may induce bystander senescence in non‐CMV specific T cells (Fletcher et al., 2005). Therefore the cytotoxic CD8^+^ T_sen_ populations that accumulate during ageing and during persistent viral infections are now re‐focussed to preferentially recognize NK ligands that may be expressed by infected, malignant or “stressed” tissues (Pereira et al., 2019; Sagiv et al., 2016). Therefore, T_sen_ can mediate NKR dependent cytotoxicity, independently of their antigen specificity.

## 4 THE KILLING OF SENESCENT NON‐LYMPHOID TISSUE CELLS BY NK AND CD8^+^ T CELLS

Senescent non‐lymphoid cells accumulate in many tissues during ageing (Campisi, 2013) and are associated with tissue dysfunction that lead to frailty. Transgenic mouse models that enable the specific removal of senescent cells from different tissues *in vivo* show that the elimination of these cells increases lifespan, improves overall fitness and reduces age‐associated characteristics of the animals (Baar et al., 2017; Baker et al., 2011, 2016). An exciting observation was that cells of the immune system, including NK, CD4^+^ and CD8^+^ T cells, can also recognize and eliminate senescent cells *in vitro* and *in vivo* (Gorgoulis et al., 2019; Pereira et al., 2019), a new role form immunosurveillance that may be relevant for ageing. The mechanisms involved in this include the interaction between NKG2D and NKG2A/C receptors on CD8^+^ T and NK cells, and their ligands such as MICA/B and HLA‐E expressed by senescent stromal cells (Krizhanovsky et al., 2008; Pereira et al., 2019; Sagiv et al., 2016).

Despite the evidence for senescent cell clearance by the immune system, it is not yet clear why senescent cells accumulate during ageing and persist at sites of age‐related pathology (Karin et al., 2019; Ovadya et al., 2018; Sharpless & Sherr, 2015). Altered immunity during ageing may contribute to this, however senescent cells also elicit evasion strategies, including the expression of inhibitory NK receptor ligands such as HLA‐E (Pereira et al., 2019) and the shedding of NKG2D ligands that act as decoy receptors that interfere with cytotoxic cell recognition and killing (Muñoz et al., 2019). Therefore a balance between activating and inhibitory signals will determine the outcome of the NK and T cell cytotoxic response to senescent tissue cells. On one hand, the development of NK‐like function in CD8^+^ T_sen_ cells that lose the capacity to proliferate may be beneficial as it enables the retention of cytotoxic cells with broad spectrum of activity during ageing that may be important for killing of infected and malignant cells. However the acquisition of NK‐like function in T cells during ageing may have negative consequences and induce pathology, for example in skin lesion formation in patients with cutaneous leishmaniasis (Figure 1).

## 5 IMMUNITY IN PATIENTS WITH CUTANEOUS LEISHMANIASIS; THE DOUBLE‐EDGED SWORD

*Leishmania braziliensis* is the main causal agent of cutaneous leishmaniasis (CL) in Brazil, where destructive cutaneous lesions develop (World Health Organization/Department of control of neglected tropical diseases, 2015). Patients with *L*.* braziliensis* skin infections usually develop lesions after 2–4 weeks (Oliveira‐Ribeiro et al., 2017). The early stages of infection do not present with CD8^+^ T cell infiltration, but CD8^+^ T cell activity increases as the infection progresses and the characteristic CL skin lesions form (Faria et al., 2009). Production of inflammatory mediators such as IFN‐γ as well as CD8^+^ cytotoxic T cell activity is observed during the acute and healing phase of *L*.* major* infection and is linked to parasite clearance (Rossi & Fasel, 2017). There is a strong correlation between the severity of the disease and the number of CD8^+^ T cells present in the lesion (Faria et al., 2009; Santos et al., 2013), but this is independent of the presence of parasites in the lesions (Carvalho et al., 2007; Murray et al., 2005; Pearson et al., 1996). Despite this, if untreated, the lesions increase in size progressively causing the characteristic pathology of CL. This raises the question as to the cause of the lesions in the skin suggesting the possibility that chronic inflammation and non‐specific CD8^+^ T or NK cell cytotoxic responses may lead to non‐specific tissue destruction.

*Leishmania braziliensis* infected individuals have elevated numbers of circulating CD4^+^ and CD8^+^ T cells that possess short telomeres, decreased expression of the catalytic component of the enzyme telomerase (hTERT), have reduced proliferative activity and increased expression of the nuclear DNA‐damage response protein (Covre et al., 2019). In addition, these T cells secrete SASP‐like cytokines in response to *L*.* braziliensis* antigen (LbAg) or anti‐CD3 stimulation compared to controls *in vitro*. Although T_sen_ accumulate in patients (Covre et al., 2019), the robust proliferative response to LbAg stimulation indicates that not all the *L*.* braziliensis* specific T cells that are present are senescent (Covre et al., 2019). Nevertheless, the association between the frequency of both CD4^+^ and CD8^+^ T_sen_ cells in the circulation and cutaneous lesion size in infected patients suggests that these cells may be recruited into the lesions and may contribute to the observed skin ulceration.

An interesting observation is that the accumulation of senescent T cells, but not NK correlates with the age of CL patients (Covre L and Gomes D, unpublished data). Compared to young subjects, elderly patients displayed larger cutaneous lesions, longer duration of illness and were more likely to develop mucocutaneous leishmaniasis, the most inflammatory and severe clinical form of the tegumentary leishmaniasis (Carvalho et al., 2015). Senescent T cells from CL patients but not healthy controls also upregulate the skin homing receptor CLA in response to activation with *L*.* braziliensis* antigens (Covre et al., 2019) This facilitates their homing into the skin where they can perform effector functions. Interestingly, while NK cells in the peripheral blood of have a greater cytotoxic potential compared to CD8^+^ T_sen_, NK cells have reduced CLA expression and do not home to skin lesions as efficiently as CD8^+^ T_sen_. This suggests that during acute infection with *L*.* braziliensis*, T_sen_ within both CD4^+^ and CD8^+^ subsets home to the skin where they initially control the infection. However their potent inflammatory activity may also contribute to development of the skin pathology of CL even after the parasites have been cleared. This raises the question about the mechanism involved in the tissue damage within the cutaneous lesions of these patients.

## 6 HYPOTHESIS: THE NON‐SPECIFIC NATURE OF CD8^+^ T_sen_ INDUCES IMMUNOPATHOLOGY DURING INFECTIONS SUCH AS CUTANEOUS LEISHMANIASIS

We hypothesize that dermal macrophages infected by Leishmania initiate an inflammatory response that preferentially attracts *L*.* braziliensis* specific CD8^+^ T_sen_ from the blood which clear the infection. However, the inflammation in the skin, induced by the interaction between infected macrophages and the infiltrating T cells, induces the expression of stress ligands including those that bind to NK receptors by the surrounding stroma (Gasser et al., 2005; Groh et al., 2001; Molinero et al., 2004). The interaction between these ligands and NK receptors on T_sen_ leads to cytotoxic killing and tissue damage. In turn, this damage leads to exacerbated inflammation, inducing further expression of stress ligands and results in more destruction by CD8^+^ T_sen._ This generates a destructive positive feedback loop that leads to progressively larger skin lesions if left untreated. At this advanced stage of the infection, CD8^+^ T_sen_ cells that infiltrate the inflamed lesion do not have to be specific for *L*.* braziliensis* but may be specific for other antigens (e.g. CMV) that have been shown to drive T cell senescence and therefore the acquisition of NK characteristics (Figure 1). This is supported by previous observations that the bystander activation of memory CD8^+^ T cells in the skin may contribute to the chronic inflammation and tissue damage in an NKG2D‐dependent manner during murine cutaneous leishmaniasis (Crosby et al., 2014). Furthermore, the pathogenesis of murine cutaneous leishmaniasis is exacerbated by LCMV infection‐induced CD8^+^NKG2D^+^ T cells, supporting the possibility that tissue damage can occur independently of the antigen specificity of the infiltrating cells (Crosby et al., 2015). Interestingly, human lesional CD4^+^ and CD8^+^ T cells are able to proliferate and produce IFN‐γ in response not only to Leishmania antigens but also to other pathogens (Da‐Cruz et al., 2010). This highlights that not all the T cell in the lesions are senescent but supports the role of T cells of different specificities in the promotion of cutaneous lesions in CL.

## 7 FUTURE PERSPECTIVES

The investigation of the cellular infiltrate in patients with cutaneous leishmaniasis by gene expression (Amorim et al., 2019; Christensen et al., 2016) and histological analyses is essential to determine the presence of alternative ligands for CD8^+^ T_sen_ within the tissue. It is also crucial to identify the source of inflammation at these sites, are the myeloid cells, the stromal cells, the leukocytes or all of them involved? The hypothetical positive feedback loop described here that involves T_sen_ and excessive inflammation and non‐specific tissue damage may also be involved in other non‐resolving inflammatory diseases where accumulation of T_sen_ cells have been demonstrated including Chagas disease (Molina & Kierszenbaum, 1989) and malaria (Cockburn et al., 2014). The accumulation of senescent T cells with increased pro‐inflammatory potential has been implicated during age‐related diseases such as rheumatoid arthritis (Goronzy et al., 2013; Weyand et al., 2017), Alzheimer's (Gate et al., 2020), and cardiovascular diseases (Youn et al., 2019; Yu et al., 2015), where the non‐specific tissue damage driven by T_sen_ cells needs to be investigated and might offer new opportunities for prevention and treatment (Andersson et al., 2011). Furthermore, it would important to know if inflammation that is induced by infections such as SARS‐CoV‐2 that have a disproportionate pathological impact in older individuals (Merad & Martin, 2020) results from non–specific T_sen_ recruitment and induction of non‐antigen specific damage in the lung and other tissues. Furthermore, it is currently unclear how senolytic drugs that are being used to clear senescent cells will affect senescent leukocyte populations and if this may have beneficial or detrimental consequences. Collectively we suggest that T cells that have been driven to extreme differentiation and senescence by infectious agents acquire potent MHC‐I‐unrestricted cytotoxic and capacity for inflammatory cytokine secretion together with the ability to cause non‐specific NK‐related damage. Given the increased burden of tumours and infections with age, the expansion of NK cell‐like functions in CD8^+^ T cells could be an advantageous adaptation that would enable the recognition and elimination of infected and transformed cells. However, the accumulation of senescent T cells during ageing is a double‐edged sword that may induce pathology, especially in inflammatory environments.

## CONFLICT OF INTEREST

None declared.

## AUTHORS' CONTRIBUTIONS

All four authors conceived and wrote the manuscript.
